# The Position of Nurses in Great Britain: Is the Government Partially Responsible?

**Published:** 1919-03-15

**Authors:** 


### THE EDITOR'S LETTER-BOX—(*continued*).

### The Position of Nurses in Great Britain: Is the Government Partially Responsible?

*To the Editor of* The Hospital.

Sir,—Your correspondent "Ierne," when she suggests appealing to the House of Commons for better pay and shorter working hours for nurses forgets that the Government are partially to blame for the present unsatisfactory conditions.

If it were announced that from a certain date the laws regulating the hours of work for women employed in factories, shops, and offices would be abolished, there would be a general outcry against surrendering female employees to the mercy of possibly unscrupulous employers.

Yet the Government proposes to establish a Ministry of Health, for the administration of which it will be largely dependent upon women who earn their livelihood by nursing, and apparently it is taking no steps to safeguard the interests of its employees.

Every nurse working in a hospital, a nursing home, or a private house is at the mercy of her employer. She has no fixed status: she is betwixt and between. She can call upon no powerful trade union, neither has she as yet an equivalent to the British Medical Association.

The lot of the district nurse is even less enviable: at the end of a heavy day's work she may find a note pushed under her door calling her to some remote part of her district; she may be up half the night, and yet there can be no abatement in the next day's routine. Public opinion in Great Britain trends towards conservatism, but if the present state of affairs is once realised, will it be considered desirable, or even possible, to build up the health of the nation at so high a cost?

On whom will be thrown the onus of existing conditions?

Yours faithfully,

G. M. E. L.

### THE FACTORY NURSE'S SALARY.

*To the Editor of* The Hospital.

